# Effect of pH and Temperature on the Surface Roughness of 3D-Printed and Milled Dental Hybrid Resin–Ceramic

**DOI:** 10.3390/polym17243308

**Published:** 2025-12-14

**Authors:** Seelassaya Leelaponglit, Awiruth Klaisiri, Chayanit Angkananuwat, Nantawan Krajangta

**Affiliations:** Division of Restorative Dentistry, Faculty of Dentistry, Thammasat University, Pathum Thani 12120, Thailand; seelassa@tu.ac.th (S.L.); dentton@tu.ac.th (A.K.);

**Keywords:** pH, temperature, hybrid resin ceramic, surface roughness

## Abstract

Chemical and thermal shifts in the oral cavity can damage the surface of 3D-printed hybrid resin–ceramic materials, and research on these effects is still limited. This study investigated the effects of pH and temperature variations on the surface roughness (Ra) of two milled materials, a resin nanoceramic (Cerasmart^®^; CS) and a polymer-infiltrated ceramic network (Vita Enamic^®^; EN), and a 3D-printed (VarseoSmile Crown plus^®^; VS) material. A total of 135 rectangular specimens (12 × 14 × 2 mm), 45 per material, were aged for 30 days under acidic (pH 5), alkaline (pH 9), cold (5 °C), and hot (60 °C) conditions, with neutral (pH 7, 37 °C) as a control. Ra was measured before and after aging using an optical micro-coordinate system. Two-way ANOVA and Tukey’s test assessed the effects of material type and aging condition. Paired *t*-tests evaluated changes over time. Variations in pH did not significantly increase Ra for any materials. Cold and hot temperatures increased Ra for the milled materials (*p* < 0.001). VS showed greater stability than the milled materials (CS and EN) despite its higher Ra both before and after aging under all conditions. All Ra values remained below the clinical threshold for biofilm accumulation (0.2 µm) under all conditions.

## 1. Introduction

Hybrid resin–ceramic materials are an emerging class of CAD/CAM restorative materials designed to combine the esthetic and mechanical advantages of ceramics with the flexibility and ease of the fabrication of resin composites. Compared with conventional ceramics, they exhibit reduced brittleness, improved machinability, and enhanced stress distribution, making them suitable for minimally invasive indirect restorations [1,2].

Traditionally, hybrid resin–ceramic materials were primarily fabricated through subtractive manufacturing (milling), which involves cutting pre-polymerized, highly cross-linked blocks to the desired shape. Milled examples include Cerasmart^®^ (GC Dental Products, Tokyo, Japan), a resin nanoceramic (RNC) composed of nanofiller particles in a resin matrix, and Vita Enamic^®^ (Vita Zahnfabrik, Bad Säckingen, Germany), which is a polymer-infiltrated ceramic network (PICN). The milling process yields dense, homogeneous microstructures but may introduce surface microcracks or tool-induced defects that affect surface smoothness and long-term performance [1,3,4].

The emergence of additive manufacturing (3D printing) represents a significant paradigm shift in the fabrication of hybrid resin–ceramic restorations. This technological evolution is rapidly transforming clinical dentistry by enabling superior design flexibility, markedly reduced material waste, and precise layer-by-layer polymerization. Recent innovations defining 3D printing practice around 2025 include Continuous Digital Light Processing (cDLP) for enhanced productivity, Artificial Intelligence (AI)-driven algorithms that optimize restoration geometry and mitigate the staircase effect, and sustained material science innovation in specialized resin–ceramic hybrid formulations [1]. VarseoSmile Crown plus^®^ (BEGO, Bremen, Germany) is claimed to be the first hybrid resin–ceramic specifically developed for the 3D printing of permanent single restorations. Unlike milling, 3D printing constructs the restoration by light-curing a liquid resin–ceramic suspension. This difference in fabrication leads to distinct polymerization kinetics, filler distribution, interfacial bonding, and network crosslinking density, which may alter surface hardness, topography, and resistance to chemical or thermal degradation [1,3,4].

Surface smoothness is a key determinant of clinical success, as it affects esthetics, wear resistance, and microbial adhesion. Rough surfaces promote bacterial retention and biofilm formation, increasing the risk of secondary caries, gingival inflammation, and surface discoloration [5,6,7,8]. Bollen et al. (1997) established a threshold of 0.2 µm surface roughness (Ra value) above which plaque accumulation significantly increased [5]. Therefore, achieving and maintaining a smooth surface finish is critical to ensure longevity and oral health [7]. The intraoral environment exposes restorative materials to chemical and thermal fluctuations that can alter their surface integrity. Dietary acids, beverages, and medications may reduce salivary pH below 5.5, whereas alkaline products can raise it above neutrality [9]. Similarly, ingesting hot and cold foods exposes restorations to temperature variations of approximately 5 °C to 55 °C [10]. Such variations can degrade the surface quality of resin-based materials, influencing roughness, wear, and biofilm accumulation [5,11,12,13,14,15,16,17]

Although numerous studies have examined the effects of pH or temperature on resin-based restorative materials [5,8,18,19,20,21] evidence for hybrid resin–ceramic, particularly 3D-printed, remains limited and inconsistent. Recent investigations comparing milled and printed hybrid resin–ceramic demonstrate that 3D-printed specimens often exhibit higher initial roughness but can approach milled surfaces after glazing or polishing; however, subsequent thermocycling may either increase or decrease Ra depending on finishing protocol [1]. Other studies emphasize processing parameters, printing orientation, layer thickness, and post-curing schedule, which significantly affect accuracy, reliability, and surface topography, indicating anisotropic and process-dependent behavior [21,22]. The evidence for milled hybrid resin–ceramic is likewise inconsistent. Some reports show that thermal or water aging reduces flexural strength while maintaining Ra values, whereas others find that strong acid or long-term immersion can increase Ra depending on filler morphology and resin content [23].

Despite these findings, there remains a distinct research gap due to the limited number of studies examining the effects of pH and temperature on hybrid resin–ceramic materials and the inconsistent results reported across the existing literature. The present study addresses this gap by evaluating three representative hybrid resin–ceramic materials fabricated through distinct manufacturing techniques, two milled, a resin nanoceramic (RNC, Cerasmart^®^) and a polymer-infiltrated ceramic network (PICN, Vita Enamic^®^), and a 3D-printed (VarseoSmile Crown plus^®^), to investigate the influence of pH and temperature variation on surface roughness under standardized 30 days artificial aging conditions. The following null hypotheses were tested: The first null hypothesis was that artificial aging under acidic, neutral, and alkaline pH conditions would not significantly affect surface roughness. The second null hypothesis stated that artificial aging under cold, body, and hot temperature conditions does not significantly affect surface roughness. The third null hypothesis was that there would be no difference in surface roughness changes between milled and 3D-printed hybrid resin–ceramic materials under any aging condition.

## 2. Materials and Methods

### 2.1. Study Design

**Figure 1:**
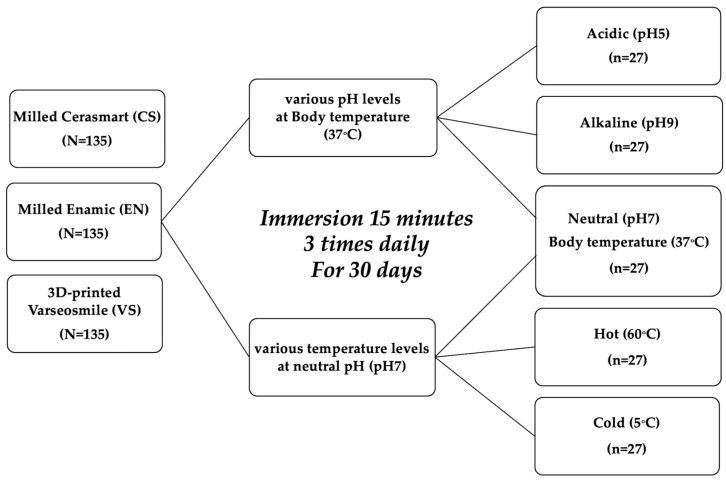
Study design.

This study evaluated the effects of pH and temperature on the surface roughness of 3D-printed and milled resin–ceramic hybrid materials. Two milled hybrid materials (Cerasmart^®^: CS and Vita Enamic^®^: EN) and one 3D-printed hybrid material (VarseoSmile Crown plus^®^: VS) were subjected to five aging conditions: two subgroups of different pH values (acidic pH 5 and alkaline pH 9), two subgroups of temperature (hot 60 °C and cold 5 °C), and neutral with body temperature as a control group (Figure 1).

Sample size calculation using G*Power software version 3.1.9.7 (effect size = 0.10, α = 0.05, power > 0.95) indicated that 27 specimens per subgroup were required, yielding a total of 135 specimens per material. Specimens from each material type were randomly allocated to the study subgroups, and the baseline surface roughness values were statistically verified to ensure homogeneity across groups prior to artificial aging. To minimize bias, the operator performing the surface roughness measurements was blinded to both the material type and aging condition, ensuring a blind experimental design.

### 2.2. Specimen Preparation

**Figure 2:**
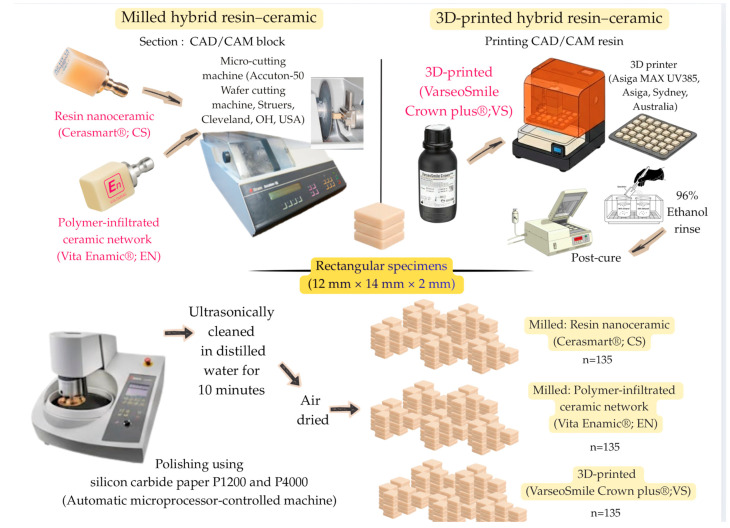
Specimen preparation.

A total of 135 rectangular specimens (12 mm × 14 mm × 2 mm) were prepared for each hybrid resin–ceramic material (Figure 2). For the milled groups, Cerasmart^®^ (CS; GC Dental Products, Tokyo, Japan) and Vita Enamic^®^ (EN; Vita Zahnfabrik, Bad Säckingen, Germany) specimens were sectioned from prefabricated CAD/CAM blocks using a precision micro-cutting machine (Accuton-50 Wafer cutting machine, Struers, Cleveland, OH, USA) under constant water cooling. For the 3D-printed group, VarseoSmile Crown plus^®^ (VS; BEGO, Bremen, Germany) specimens were designed using 3D modeling software and fabricated using a digital light processing (DLP) 3D printer (Asiga MAX UV385, Asiga, Sydney, Australia) equipped with a 385 nm ultraviolet light-emitting diode. After printing, the uncured resin was removed by immersion in 96% ethanol for 3 min, followed by a second rinse in fresh 96% ethanol for 2 min to ensure complete removal of residual monomers. Each specimen was then postcured for 2 × 1000 flashes using a xenon light polymerization unit (Otoflash G171, NK-Optik, Baierbrunn, Germany) under a nitrogen atmosphere.

All specimens were polished using an automatic microprocessor-controlled machine for grinding and polishing (Struers, Cleveland, OH, USA) with sequential use of silicon carbide paper P1200 and P4000 (Water Proof SiC Paper; Struers, Cleveland, OH, USA) under running water with 5 N pressure at 300 rpm for the polishing disk and co-rotation at 150 rpm for 20 s. Following polishing, the specimens were ultrasonically cleaned in distilled water for 10 min and air-dried before baseline measurements.

### 2.3. Artificial Saliva Preparation

The artificial saliva formulation followed the procedure described in a previous study [24]. It consisted of 2.0 g L^−1^ methyl-p-hydroxybenzoate, 0.625 g L^−1^ potassium chloride (KCl), 0.059 g L^−1^ magnesium chloride hexahydrate (MgCl_2_·6H_2_O), 0.166 g L^−1^ calcium chloride dihydrate (CaCl_2_·2H_2_O), 0.804 g L^−1^ dipotassium hydrogen phosphate (K_2_HPO_4_), 0.326 g L^−1^ potassium dihydrogen phosphate (KH_2_PO_4_), and 10.0 g L^−1^ sodium carboxymethyl cellulose, with all reagents obtained from Sigma-Aldrich (St. Louis, MO, USA). Each chemical component was sequentially added to 1000 mL of deionized water and continuously stirred using a magnetic stirrer at room temperature for approximately six hours to ensure complete dissolution and homogeneity of the solution. The prepared saliva substitute was maintained at 37 °C and used as the immersion medium during the artificial aging process. To maintain chemical stability and minimize microbial contamination, the artificial saliva solutions were renewed every 24 h.

### 2.4. pH Conditions for Artificial Aging

**Figure 3:**
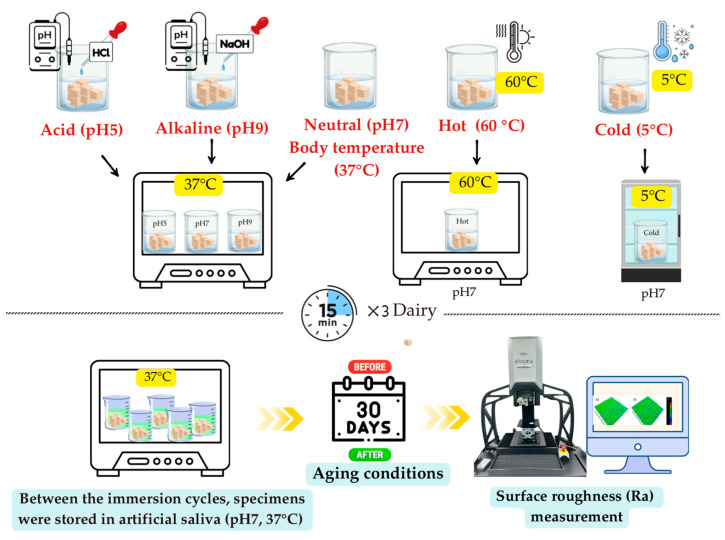
pH and temperature conditions for artificial aging.

Artificial aging of the specimens (*n* = 27) was conducted through immersion in artificial saliva solutions prepared at three different pH levels: acidic (pH 5), neutral (pH 7), and alkaline (pH 9) (Figure 3). Each subgroup was maintained at the respective pH for 15 min, three times daily (morning, afternoon, and evening), for 30 days. Between the immersion cycles, the specimens were stored in artificial saliva at a constant temperature of 37 °C and neutral pH within the incubator.

For acidic conditions (pH 5), hydrochloric acid (HCl) was added incrementally until the desired value was reached. The neutral condition (pH 7) required no modification because it corresponded to the natural pH of the prepared medium. Under alkaline conditions (pH 9), sodium hydroxide (NaOH) was added dropwise with continuous pH monitoring to achieve the target value. The pH of each solution was adjusted using acid or base titration under continuous monitoring using a calibrated pH meter (Orion 2-Star; Thermo Fisher Scientific, Waltham, MA, USA). The pH of all solutions was verified immediately after adjustment, and daily pH measurements were recorded throughout the immersion period.

### 2.5. Temperature Conditions for Artificial Aging

Artificial aging of the specimens (n = 27) was conducted through immersion in artificial saliva solutions at hot (60 °C), body temperature (37 °C), and cold (5 °C) conditions (Figure 3). Each subgroup was maintained at the respective temperature for 15 min, three times daily (morning, afternoon, and evening), for 30 days. Between the immersion cycles, the specimens were stored in artificial saliva at a constant temperature of 37 °C and neutral pH within the incubator.

The specimen container for the group exposed to cold (5 °C) temperature was placed inside a refrigerator (Artiko, Esbjerg, Denmark) to maintain the desired temperature. The specimen containers for the groups exposed to body temperature (37 °C) and hot (60 °C) conditions were placed in an incubator (Humanlab, Gyeongqi-Do, Republic of Korea). To maintain the temperature required for immersion, a digital thermometer was used to continuously monitor the temperature of the solutions throughout the aging period.

### 2.6. Surface Roughness Measurement

Surface roughness (Ra) was measured before and after 30 days of artificial aging using an Alicona InfiniteFocus^®^ G6 optical micro-coordinate measurement system (µCMM; Alicona Imaging GmbH, Raaba/Graz, Austria). All measurements were conducted by a single, trained operator following the associated software protocol (MeasureSuite v.5.3.1; Alicona Imaging GmbH). To minimize bias, a double-blind design was employed, where both the operator and the statistician were blinded to the material types.

### 2.7. Statistical Analysis

Statistical analyses were performed using SPSS (Version 27; IBM Corporation, Armonk, NY, USA), with a significance level set at α = 0.05. Descriptive statistics, including mean and standard deviation, were calculated. Data normality (*p* > 0.05), assessed for each subgroup using the Shapiro–Wilk test, and homogeneity of variances (*p* > 0.05), assessed using Levene’s test, were both confirmed, validating the use of parametric analyses.

A two-way analysis of variance (ANOVA) and Tukey’s HSD post hoc test were used to evaluate the effects of the three material types and five aging conditions on surface roughness. Paired *t*-tests were employed to assess the changes in surface roughness from before to after aging of each specimen within each material group.

## 3. Results

Two-way ANOVA revealed significant differences in surface roughness (Ra value) across material types and aging conditions. A significant interaction effect indicated that the influence of aging conditions on surface roughness varied depending on the material type.

The mean Ra values, standard deviations (SDs), and specific statistical analyses for the hybrid resin–ceramic materials following 30 days of artificial aging at various pH levels are presented in Table 1. The results for aging at different temperature levels are presented in Table 2.

**Figure 4:**
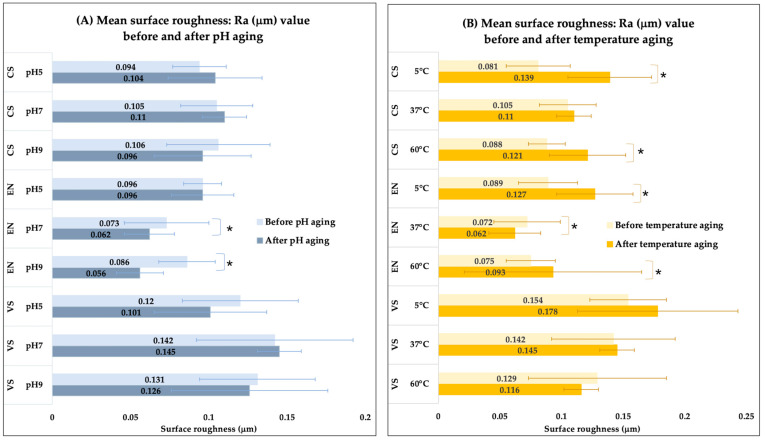
Bar graphs represent surface roughness (Ra) values for the three hybrid resin–ceramic materials before and after 30 days of artificial aging. (**A**) Effects of different pH levels (pH 5, 7, 9) and (**B**) effects of different temperatures (5 °C, 37 °C, 60 °C). An asterisk (*) indicates a significant difference between the “before” and “after” measurement within the same material and condition.

Across all tested conditions, VarseoSmile Crown plus^®^ (VS) consistently exhibited higher baseline Ra values (ranging from 0.120 to 0.154 µm) compared to both milled materials: Cerasmart^®^ (CS) (ranging from 0.081 to 0.106 µm) and Vita Enamic^®^ (EN) (ranging from 0.072 to 0.096 µm). This pattern persisted both before and after artificial aging (Figure 4).

## 4. Discussion

Hybrid resin–ceramic materials, such as Cerasmart^®^ (CS), Vita Enamic^®^ (EN), and VarseoSmile Crown plus^®^ (VS), represent an advanced class of restorative materials that merge the favorable mechanical, esthetic, and handling properties of both ceramic and resin-based systems. Their microstructure, comprising ceramic fillers within a resin matrix or an interpenetrating polymer–ceramic network, offers a promising balance between strength and flexibility, making them suitable for chairside CAD/CAM restorations. However, the long-term clinical success of these materials is contingent upon their resistance to degradation under common intraoral challenges, particularly variations in pH and temperature, which are known to influence surface integrity and performance over time. Although intraoral aging involves complex, multifactorial processes that vary with patient habits, dietary exposure, and salivary buffering capacity, controlled static immersion in simulated aging solutions provides standardized acceleration of chemical breakdown processes that occur gradually in vivo. In the present study, the surface roughness of two milled materials (CS and EN) and one 3D-printed (VS) hybrid resin–ceramic material was evaluated following a 30-day artificial aging protocol under varying pH and temperature conditions. This immersion duration is consistent with prior studies assessing the durability of CAD/CAM resin-based materials and is considered to simulate several months to over a year of clinical service, depending on the nature and frequency of environmental exposure [6,7,8].

The first null hypothesis, stating that artificial aging under varying pH conditions would not significantly affect surface roughness, must be rejected. The results show that while some material condition combinations remained stable, significant changes occurred in specific groups. EN, a milled material, demonstrated a significant decrease in surface roughness at both pH 7 and pH 9. This smoothing effect under neutral to alkaline conditions suggests that surface degradation or etching did not occur; instead, potential surface polishing or loss of a minor component may have occurred. In contrast, CS and VS maintained generally stable Ra values across all pH levels, suggesting a higher degree of chemical stability within this pH range.

The second null hypothesis, which proposed no significant effect of temperature conditions on surface roughness, must also be rejected. Temperature variations caused more widespread changes than pH variations. Notably, both CS and EN exhibited significant increases in surface roughness at the extreme temperature conditions (5 °C and 60 °C). This suggests that thermal stress or accelerated degradation mechanisms are active at temperatures deviating from body temperature (37 °C). The stability observed at 37 °C for most materials indicates that all tested materials perform predictably under normal oral cavity temperature conditions. The significant increase in Ra at lower and higher temperatures suggests potential material property changes that compromise surface integrity.

The third null hypothesis, stating no difference in surface roughness changes between material processing methods, is rejected based on the significant interaction effect observed in the results within Table 1 and Table 2. The 3D-printed material, VS, consistently demonstrated the highest baseline surface roughness values across all conditions compared to the milled materials (CS and EN).

CS exhibited favorable surface stability when subjected to varying pH challenges (pH 5, 7, and 9); however, CS demonstrated increased surface roughness following exposure to temperature extremes (5 °C and 60 °C). This differential response can be explained by investigating its composition and the mechanism of material degradation. CS is classified as a resin nanoceramic (RNC), with dispersed fillers, that incorporates approximately 71% of silica and barium glass nanoparticles within a UDMA-rich resin matrix [9]. The discrete ceramic particles are individually encapsulated by silane coupling agents, creating effective hydrolytic barriers around each filler particle. The surface stability of CS under various pH conditions can be attributed to several factors. First, the dispersed nanofiller structure minimizes continuous polymer exposure to pH challenges [9]. Second, the UDMA-rich polymer matrix demonstrates enhanced hydrolytic stability compared to TEGDMA-dominant systems due to urethane linkages that resist hydrolytic degradation [10]. Additionally, proprietary filler treatments with effective silane bonding create hydrophobic interfaces that further resist water sorption and subsequent chemical degradation [10]. This enhanced integration may prevent the penetration of hydrogen ions and hydroxyl groups that typically catalyze degradation processes under acidic or alkaline conditions. However, the increased surface roughness of CS observed after exposure to temperature extremes suggests that these interfaces may be particularly susceptible to thermally induced degradation mechanisms. In these conditions, the differential coefficient of thermal expansion (CTE) between organic and inorganic components generates significant localized stresses at the filler–matrix boundaries [11]. Silica and barium glass nanoparticle fillers have a low CTE (0.5–1 × 10^−6^/°C), whereas the resin matrix, typically composed of UDMA and other monomers, has a higher CTE (50–70 × 10^−6^/°C) [11]. This mismatch between the filler and matrix CTEs can result in dimensional changes and potential degradation under thermal stress. Thermally induced swelling and partial degradation of the polymer matrix can compromise surface smoothness and lead to microcrack formation [11].

Among the three materials tested, EN was the only one to show a statistically significant reduction in surface roughness after immersion in neutral (pH 7) and alkaline (pH 9) conditions. EN is classified as a polymer-infiltrated ceramic network (PICN) material that comprises a feldspathic ceramic network (86 wt%) interpenetrated by an organic polymer network (14 wt%). The ceramic component, primarily feldspathic ceramic, consists of silicon dioxide (SiO_2_), aluminum oxide (Al_2_O_3_), sodium oxide (Na_2_O), and potassium oxide (K_2_O), while the polymer phase incorporates urethane dimethacrylate (UDMA) and triethylene glycol dimethacrylate (TEGDMA) [12]. This surface smoothening (reduction in Ra) in neutral and alkaline environments is a unique and notable finding compared to CS and VS, offering a novel mechanistic perspective on the surface stability of PICN materials under chemical stress. EN contains interconnected ceramic pores infiltrated with polymers, creating numerous interfaces where polymer protrusion may occur [13]. Those elevated polymer domains may exhibit heightened susceptibility to chemical degradation, and their preferential dissolution during aging promotes the development of a more uniform and smoother surface profile [14]. This pH-driven process likely involves hydrolytic dissolution of the less stable polymer-rich components at the outermost layer of the interpenetrating network, producing a “polishing” effect by eliminating the most exposed organic material. Additionally, Alnasser et al. (2019) and Hussein et al. (2020) found that the organic polymer network of restorative materials can absorb water and other substances from the environment, leading to swelling of the polymer matrix, which can result in smoother surface roughness under all pH conditions [15,16]. The observed reduction in surface roughness of EN following pH exposure must be interpreted with considerable caution. While a smoother surface may appear favorable, this phenomenon reflects superficial dissolution of the polymer-rich domains at the material surface rather than an intrinsic improvement in surface quality. This process may compromise the long-term durability of the restoration by reducing the polymer–ceramic interpenetration zone that contributes to mechanical reinforcement. Furthermore, the leaching or erosion of polymer components could predispose the material to microcrack formation, discoloration, and increased water sorption over time, potentially undermining clinical longevity [16]. Conversely, thermal challenges, particularly at the extremes (5 °C and 60 °C), create differential expansion and contraction between the ceramic and polymer phases due to their inherently dissimilar coefficients of thermal expansion (CTE). Feldspathic ceramic has a low CTE (~8–10 × 10^−6^/°C), while the polymer phase exhibits a higher CTE (~50–70 × 10^−6^/°C) [15]. This significant disparity can lead to internal stresses during thermal cycling, potentially causing microcracks or interfacial debonding and consequent increases in surface roughness [11,16].

In contrast to the milled materials, VS demonstrated exceptional stability by maintaining consistent surface characteristics when subjected to both pH variations and thermal challenges. VS is a 3D-printed hybrid material incorporating 30–50% silanized ceramic fillers within a highly cross-linked resin matrix composed of esterification products of 4,4’-isopropylidiphenol and methacrylates [21]. This dual-phase structure confers enhanced hydrolysis resistance by significantly limiting water sorption (<12 µg/mm^3^) and solubility (<1 µg/mm^3^), properties that are critical for maintaining stability across varying pH environments, as reported by the manufacturer [18]. Additionally, the material incorporates advanced functional monomers, such as diphenyl phosphine oxide photoinitiators, which enhance cross-linking density and further minimize chain mobility and water penetration, creating a more hydrolysis-resistant interface between organic and inorganic components [20]. The enhanced thermal stability observed in VS is due to several material composition and manufacturing-related factors. The ceramic fillers in VS are silanized and embedded within a highly cross-linked polymer matrix, which may reduce the disparity in thermal expansion between organic and inorganic components. The light-activated resin formulation that includes diphenyl phosphine oxide photoinitiators and bisphenol-derived methacrylates can promote high cross-link density, limit chain mobility, and increase dimensional stability [19]. Furthermore, the additive manufacturing process of VS allows for a more homogeneous distribution of fillers and a reduction in interfacial defects, which contributes to its thermal stability. The limited CTE mismatch between the matrix and fillers, combined with the high cross-link density, mitigates thermally induced dimensional stress and preserves surface integrity. These material features position VS as a promising candidate for restorations subjected to frequent thermal and chemical fluctuations in the oral cavity.

Despite demonstrating greater surface stability after artificial aging, VS exhibited consistently higher baseline surface roughness compared to the milled hybrid resin–ceramic materials, CS and EN, across all tested conditions. This observation can be explained by the fundamental differences in material composition, polymer structure, filler content, and manufacturing process between 3D-printed and milled CAD/CAM materials. VS is fabricated through additive manufacturing using digital light processing (DLP), which involves the polymerization of a photoreactive resin in successive layers. The layer-by-layer nature of DLP printing inherently produces difficult-to-remove defects, like staircase artifacts and interlayer lines, despite the technology’s overall dimensional accuracy [21]. As a result, even after polishing, the printed material may retain microscale undulations and residual texture, contributing to higher measured surface roughness compared to homogeneously polymerized, industrially milled blocks. In contrast, CS and EN are produced under high-pressure, high-temperature polymerization, which yields a more densely cured and smooth industrial surface, particularly at the filler–matrix interface.

The comparative findings in this study have important clinical implications, particularly for the use of rapidly advancing 3D printing technologies in restorative dentistry. Even though the surface roughness values for all experimental groups remained below the established biofilm accumulation threshold of Ra < 0.2 µm, the degree of surface change differed by material type and by the specific chemical or thermal exposure. The milled groups, CS and EN, showed marked vulnerability to thermal stress, suggesting caution when treating individuals who frequently alternate between very hot and very cold beverages. In contrast, the printed material, VS, demonstrated strong stability across all conditions, indicating greater resilience in demanding environments. Despite this advantage, the 3D-printed material consistently exhibited a rougher surface than the milled groups, particularly in its initial state, creating an important clinical trade-off. The printed material is highly stable during aging, but its DLP-inherent surface texture requires careful postprocessing and well-refined polishing protocols to limit early biofilm retention and maintain esthetics. Advancements in printing, including finer layer resolution and AI-optimized print paths, will be essential to reduce the initial surface quality gap between printed and milled materials while preserving the strong material stability shown in this study. As a result, selecting an appropriate material requires balancing long-term durability with inherent surface characteristics to achieve the best clinical outcomes.

The 30-day artificial aging protocol provided a valuable basis for comparative evaluation; however, it did not fully replicate the multifactorial nature of intraoral aging. These findings require cautious interpretation, as the oral environment is a complex, dynamic system where intermittent exposure, mechanical stresses (such as mastication and tooth brushing), salivary flow, and microbial biofilm activity can interact to influence material degradation over time. This study primarily focused on surface roughness, a clinically relevant parameter. However, pH and temperature effects were evaluated separately to isolate their individual contributions. The potential combined or interactive influence of these factors on hybrid resin–ceramic materials, particularly across milled and 3D-printed systems, remains unexplored and represents an important direction for future research. Further investigation into microstructural changes using techniques such as scanning electron microscopy (SEM) and atomic force microscopy (AFM) is recommended. Clinical use of these restorative materials should involve periodic follow-up assessments to monitor restorations, with polishing or minor repairs performed as needed to prevent functional or esthetic failure. Future studies should incorporate more comprehensive aging protocols, including mechanical loading, thermocycling, pH cycling, and biofilm exposure, to better simulate clinical conditions. Additionally, research evaluating complementary material properties, such as microhardness, flexural strength, wear resistance, and color stability, is warranted to guide evidence-based material selection and ensure long-term restorative success. Finally, polishing and surface maintenance protocols should be assessed to determine their effectiveness in preserving surface integrity.

## 5. Conclusions

1. The effects of pH and temperature variation on artificial aging conditions were found to vary depending on the type of hybrid resin–ceramic material.
2. Vita Enamic^®^ demonstrated increased smoothing (decreased Ra) in neutral and alkaline pH environments, while Cerasmart^®^ and Vita Enamic^®^ became significantly rougher at temperature extremes (5 °C and 60 °C).
3. The 3D-printed material (VarseoSmile Crown plus^®^) consistently exhibited a higher baseline surface roughness than the milled materials; however, it displayed greater stability (no significant changes) across all tested pH and temperature conditions.
4. Material processing methods (milled and 3D printing) influence both the initial surface finish and the long-term stability under environmental stress.
5. All materials demonstrated adequate surface stability when aged at body temperature (37 °C) and neutral pH (pH 7), suggesting predictable clinical performance under normal oral conditions.

## Figures and Tables

**Table 1:** Mean ± standard deviation of surface roughness (Ra) of hybrid resin–ceramic materials before and 30 days after artificial aging at different pH levels.

Material	pH Level	Surface Roughness, Ra (µm)						*p*-Value of Paired *t*-Test
		Before Aging			After Aging			
Cerasmart^®^ (CS)	pH 5	0.094 ± 0.017	AB	a	0.104 ± 0.030	AB	a	0.165
	pH 7	0.105 ± 0.023	B	a	0.110 ± 0.014	AB	a	0.326
	pH 9	0.106 ± 0.033	B	a	0.096 ± 0.031	A	a	0.170
Vita Enamic^®^ (EN)	pH 5	0.096 ± 0.012	A	a	0.096 ± 0.020	A	a	0.948
	pH 7	0.073 ± 0.027	AB	a	0.062 ± 0.016	C	b	0.007
	pH 9	0.086 ± 0.018	AB	a	0.056 ± 0.015	C	b	<0.001
VarseoSmile Crown plus^®^ (VS)	pH 5	0.120 ± 0.037	C	a	0.101 ± 0.036	A	a	0.057
	pH 7	0.142 ± 0.050	C	a	0.145 ± 0.014	D	a	0.799
	pH 9	0.131 ± 0.037	C	a	0.126 ± 0.050	D	a	0.633

Different uppercase letters (A–D) within a column indicate significant differences among material and pH groups (two-way ANOVA with Tukey’s HSD post hoc). Different lowercase letters (a, b) within a row indicate significant differences between “before” and “after” aging (paired *t*-tests).

**Table 2:** Mean ± standard deviation of surface roughness (Ra) of hybrid resin–ceramic materials before and 30 days after artificial aging at different temperature levels.

Material	Temperature Level	Surface Roughness, Ra (µm)						*p*-Value of Paired *t*-Test
		Before Aging			After Aging			
Cerasmart^®^ (CS)	5 °C	0.081 ± 0.021	AB	a	0.139 ± 0.034	AE	b	<0.001
	37 °C	0.105 ± 0.023	B	a	0.110 ± 0.014	BC	a	0.326
	60 °C	0.088 ± 0.015	AB	a	0.121 ± 0.031	ACE	b	<0.001
Vita Enamic^®^ (EN)	5 °C	0.089 ± 0.024	AB	a	0.127 ± 0.031	ACE	b	<0.001
	37 °C	0.072 ± 0.027	A	a	0.062 ± 0.021	D	b	0.007
	60 °C	0.075 ± 0.020	A	a	0.093 ± 0.022	B	b	<0.001
VarseoSmile Crown plus^®^ (VS)	5 °C	0.154 ± 0.031	C	a	0.178 ± 0.065	F	a	0.070
	37 °C	0.142 ± 0.050	C	a	0.145 ± 0.014	E	a	0.789
	60 °C	0.129 ± 0.056	C	a	0.116 ± 0.014	BCA	a	0.256

Different uppercase letters (A–F) within a column indicate significant differences among material and temperature groups (two-way ANOVA with Tukey’s HSD post hoc). Different lowercase letters (a, b) within a row indicate significant differences between “before” and “after” aging measurement (paired *t*-tests).

## Data Availability

The original contributions presented in this study are included in this article. Further inquiries can be directed to the corresponding author.
